# IL13RA2 promotes progression of infantile haemangioma by activating glycolysis and the Wnt/β-catenin signaling pathway

**DOI:** 10.32604/or.2024.048315

**Published:** 2024-08-23

**Authors:** ZIYONG LIU, TAO MA, JINFANG LI, WEI REN, ZHIXIN ZHANG

**Affiliations:** 1 Department of Cardiothoracic Surgery, 970th Hospital of the People’s Liberation Army, Weihai, 264209, China; 2 Department of Cardiac Ultrasound, Weihai Municipal Hospital, Weihai, 264200, China

**Keywords:** Infantile haemangioma, IL13RA2, Glycolysis, Wnt/β-catenin pathway

## Abstract

**Background:**

Interleukin 13 receptor subunit alpha 2 (IL13RA2) plays an essential role in the progression of many cancers. However, the role of IL13RA2 in infantile haemangioma (IH) is still unknown.

**Materials and Methods:**

IL13RA2 expression in IH tissues was analyzed using western blot, qRT-PCR, and immunofluorescence. The role of IL13RA2 in haemangioma-derived endothelial cells (HemECs) was determined following knockdown or overexpression of IL13RA2 using CCK-8, colony formation, apoptosis, wound healing, tubule formation, Transwell, and western blot.

**Results:**

IL13RA2 expression was upregulated in IH tissues. IL13RA2 overexpression promoted proliferation, migration, and invasion of HemECs and induced glycolysis, which was confirmed with a glycolysis inhibitor. Specifically, IL13RA2 interacted with β-catenin and activated the Wnt/β-catenin pathway in HemECs, which were involved in the above-mentioned effects of IL13RA2.

**Conclusions:**

These findings revealed that targeting IL13RA2 is a potential therapeutic approach for IH.

## Introduction

Infantile haemangioma (IH) is a benign vascular tumors [1]. IH develops in the first few weeks after birth and proliferates rapidly in the first year of life [2]. Propranolol, a β-blocker, is the primary drug used for the treatment of IH; however, it has many side effects, such as bradycardia and hypotension [3]. The combination of β blockers and laser treatment is advantageous for the treatment of refractory haemangioma as it reduces the required dosage of oral propranolol and associated adverse reactions [4,5]. Unfortunately, due to tumor location, size, and rate of proliferation, a minority of IH cases are associated with severe complications such as disfigurement, vision loss, ulceration, and danger to lives [6]. Hence, further clarification of mechanisms underlying IH pathogenesis is important to minimize the development of IH and associated complications.

Interleukin 13 receptor subunit alpha 2 (IL13RA2) is a receptor for interleukin 13 (IL-13) [7]. Recently, IL13RA2 expression has been examined in plentiful cancers. IL-13/IL-13RA2 aggravates tumorigenesis of colorectal cancer stem cells through accelerating p53 ubiquitination [8]. IL13RA2 knockdown inhibits the migration of papillary thyroid carcinoma cells by regulating epithelial-mesenchymal transition (EMT) [9]. Additionally, IL13RA2 is regarded as an attractive therapeutic target for glioma [10]. Therefore, IL13RA2 might be a potential therapeutic target for IH.

Most tumor cells utilize glucose principally through aerobic glycolysis to meet their energy supply even with plenty of oxygen [11]. Restraint of glycolysis through targeting PFKFB3 inhibits IH progression [12]. In addition, OTUB1 regulates the angiogenesis in IH via mediating glycolysis [13]. Hence, inhibiting aerobic glycolysis might be a meritorious pattern for IH treatment.

Wnt/β-catenin pathway is activated in various malignancies and is known to potentiate tumor recurrence [14]. A previous study reported that renin acted as renin receptor to promote proliferation of IH cells via the Wnt pathway [15]. Dai et al. reported that luteolin, a flavonoid, inhibited IH through targeting Frizzled-6 via the Wnt pathway [16]. Inhibition of the Wnt/β-catenin pathway will bring about outstanding results in the treatment of IH.

In our study, IL13RA2 expression was observably up-regulated in IH. IL13RA2 was found to mediate the proliferation, migration, and invasion of haemangioma-derived endothelial cell shaemangioma (HemECs). Knockdown of IL13RA2 inhibited glycolysis and the Wnt/β-catenin pathway. Our findings suggest that IL13RA2 is a therapeutic target for IH.

## Materials and Methods

### Bioinformatics analyses

GSE43742 was obtained from the Gene Expression Omnibus. Gene Set Enrichment Analysis (GSEA) was conducted with GSE43742 using the GSEA software with hallmark gene sets.

### Tissue specimens

Four proliferating and four involuting haemangioma tissues were collected from patients. Normal group was four foreskin tissues. Written informed consent was obtained from all patients. This study was approved by the Ethics Committee of 970th Hospital of the People’s Liberation Army. All methods were performed in accordance with the ethical standards as laid down in the Declaration of Helsinki and its later amendments or comparable ethical standards.

### Isolation of endothelial cells

We established primary cultures of HemECs from four proliferating IH tissues as described previously [17]. Tissues were digested with 0.2% collagenase type 1, centrifuged, resuspended. HemECs were selectively isolated using anti-CD31-coated magnetic beads (Miltenyi Biotec, Bergisch Gladbach, Germany). HemECs were cultured in endothelial cell growth medium-2 supplemented with 20% fetal bovine serum (FBS) on fibronectin-coated plates at 37°C in a 5% CO_2_ environment.

### Immunofluorescence

Tissues and HemECs were fixed with 4% paraformaldehyde and blocked with 10% goat serum. The fixed specimens were incubated with primary antibodies overnight at 4°C: IL13RA2 (1:500, 11059-1-AP, Proteintech, Wuhan, China) α-SMA (1:800, #19245, Cell Signaling Technology, Danvers, USA), CD31 (1:800, #3528, Cell Signaling Technology), vWF antibody (1:500, ab6994, Abcam, Cambridge, UK) and β-catenin (1:500, 8480, Cell Signaling Technology). Cells were incubated with secondary antibody (1:1000, ab150077, Abcam) for 1 h. Nuclei were stained with DAPI (Santa Cruz Biotechnology, Dallas, USA). Specimens were imaged using a fluorescence microscope.

### Cell transfection and treatment

HemECs were transfected with pcDNA3.1-IL13RA2 and negative control (GenePharma, Shanghai, China) using Lipofectamine 2000 (Invitrogen, Waltham, USA). HemECs were incubated with IL13RA2-short hairpin RNA (shRNA) (GenePharma) and polybrene (6 μg/mL). After 48 h, puromycin was added to select stably transfected cells. HemECs were treated with 2-deoxy-D-glucose (2-DG) at 5 mM or XAV-939 at 10 μM for 24 h.

### qRT-PCR

Total RNA was isolated from tissues using RNA-easy isolation reagent (R701-01, Vazyme, Nanjing, China). cDNA was reversed from total RNA with HiScript® III Reverse Transcriptase (R302-01, Vazyme). qRT-PCR was performedusing ChamQ SYBR qPCR Master Mix (Q311-02, Vazyme) on CFX96 PCR system (Bio-Rad, Hercules, USA). The following primers were used: IL13RA2, forward AACAATGCTGGGAAGGTGAAGA and reverse GGGTAGGTGTTTGGCTTACG. β-actin, forward AACACCCCAGCCATGTACGTT and reverse CCATCTCTTGCTCGAAGTCCA.

### Cell viability

HemECs (2 × 10^3^/well) were seeded in 96-well plates. At 24, 48, and 72 h, 10 μL of CCK-8 (CA1210, Solarbio, Beijing, China) was added to each well for 1 h at 37°C. Cell viability was assessed by measuring the absorbance at 450 nm.

### Colony formation assay

HemECs (2000/well) were seeded in 6-well plates. 2 weeks later, the colonies were fixed with methanol, stained with crystal violet, and captured by light microscope.

### Apoptosis assay

HemECs were suspended in binding buffer and stained with Annexin V-FITC apoptosis detection kit (C1062M, Beyotime, Jiangsu, China). The rate of apoptosis was analysed by flow cytometry (BD Bioscience, Franklin Lakes, USA).

### Wound-healing assay

HemECs (3 × 10^4^/well) were seeded into 24-well plates. Wound was made in cell monolayers uisng a 10 μL pipette tip. Wound density was recorded by microscope over 24 h.

### Transwell assay

HemECs in serum-free medium were added to the upper chamber, while medium containing 10% FBS was added to the lower chamber. After incubation for 24 h, migratory and invading cells were stained with crystal violet and captured by light microscope. For invasion assay, Matrigel (BD Biosciences) was used.

### Tubules formation assay

Matrigel (BD Biosciences) was added into 96-well plate and allowed to polymerise for 20 min at 37°C. HemECs were plated on 96-well plate and incubated for 12 h. The plate was observed under a microscope.

### ATP, glucose, and lactate measurements

ATP, glucose, and lactate levels were measured using appropriate kits (BC0305, Solarbio; MAK083-1KT, Sigma-Aldrich St. Louis, USA; ab65331, Abcam). Samples were analysed using a microplate reader (Bio-Rad).

### Seahorse extracellular flux assay

Extracellular acidification rate (ECAR) and oxygen consumption rate (OCR) were detected using Seahorse XF24 analyser (Seahorse, Santa Clara, USA). For ECAR, a Seahorse XF Cell Mito Stress Test kit was used. For OCR, a Seahorse XF Glycolysis Stress Test kit (Agilent Technologies, Santa Clara, USA) was used.

### Western blot

Total protein was extracted using RIPA buffer. Nuclear and cytoplasmic fractions were extracted using a nuclear and cytoplasmic protein extraction kit (Beyotime). The proteins were separated on SDS-PAGE and transferred onto PVDF membranes. The membrane was blocked with 5% skim milk, and incubated with the following primary antibodies overnight at 4°C: IL13RA2 (1:1000, 11059-1-AP, Proteintech), Bcl-2 (1:1000, ab32124, Abcam), Bax (1:1000, #5023, Cell Signaling Technology), caspase 3 (1:1000, #9662, Cell Signaling Technology), cleaved caspase 3 (1:1000, #9661, Cell Signaling Technology), caspase 9 (1:1000, #9502, Cell Signaling Technology), cleaved caspase 9 (1:1000, #7237, Cell Signaling Technology), E-cadherin (1:1000, #3195, Cell Signaling Technology), N-cadherin (22018-1-AP, Proteintech), Snail (1:1000, #3879, Cell Signaling Technology), GLUT1 (1:1000, E-AB-31556, Elabscience, Wuhan, China), HK2 (1:1000, E-AB-14706, Elabscience), LDHA (1:1000, E-AB-70210, Elabscience), PDK1 (1:1000, ab202468, Abcam), β-catenin (1:1000, #8480, Cell Signaling Technology), c-Myc (1:1000, 10828-1-AP, Proteintech), cyclin D1 (1:1000, ab16663, Abcam), Histone3 (1:1000, ab1791, Abcam), and β-actin (1:5000, 20536-1-AP, Proteintech). The membrane was incubated with secondary antibodies (1:2000, ab6721, Abcam). Protein bands were visualised using an ECL detection Kit (Solarbio).

### Co-immunoprecipitation (Co-IP)

HEK293 cells (Pricella, China) were transfected with IL13RA2 (Flag-tagged) expression vector or β-catenin (HA-tagged) expression vector (Syngentech, Beijing, China). Protein A/G agarose beads, anti-FLAG (1:100, F7425, Sigma-Aldrich), and anti-HA (1:100, ab1424, Abcam) were added to HEK293 cell lysates. Immunoprecipitation of HemECs lysates was performed using IL13RA2 (1:50, #85677, Cell Signaling Technology) and β-catenin (1:50, #8480, Cell Signaling Technology).

### Statistical analysis

Statistical significance was calculated using one-way analysis of variance, followed by Tukey’s *post hoc* test. All statistical analyses were undertaken using Prism GraphPad 7 Software. *p* < 0.05 was considered statistically significant.

## Results

### IL13RA2 expression is upregulated in IH

**Figure 1:**
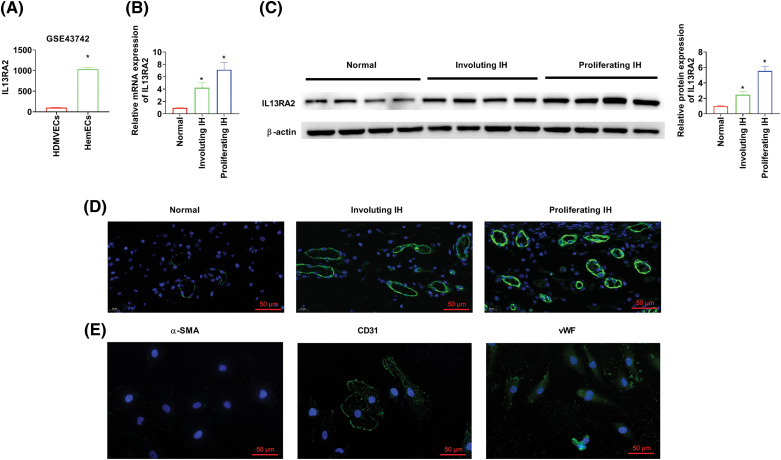
IL13RA2 expression is upregulated in IH. (A) IL13RA2 expression in HDMVEs and HemECs from patients in the GSE43742 cohort. (B–D) IL13RA2 mRNA and protein expression in tissues from patients. (E) The results of immunofluorescence staining. *p < 0.05.

We analyzed the microarray GSE43742 and found that IL13RA2 expression was remarkably higher in HemECs than in human dermal microvascular endothelial cells (HDMVECs) (Fig. 1A). Next, we evaluated IL13RA2 expression in four proliferating haemangioma tissues, four involuting haemangioma tissues, and normal tissues. IL13RA2 expression was remarkably increased in IH tissues (Figs. 1B–1D). Mesenchymal cells are often mixed with endothelial cells isolated from IH tissues. Hence, we assessed the expression of endothelial cell markers, CD31 and vWF and mesenchymal cell marker, α-SMA. Immunofluorescence staining of α-SMA, CD31, and vWF are shown in Fig. 1E.

### IL13RA2 enhances proliferation of HemECs

**Figure 2:**
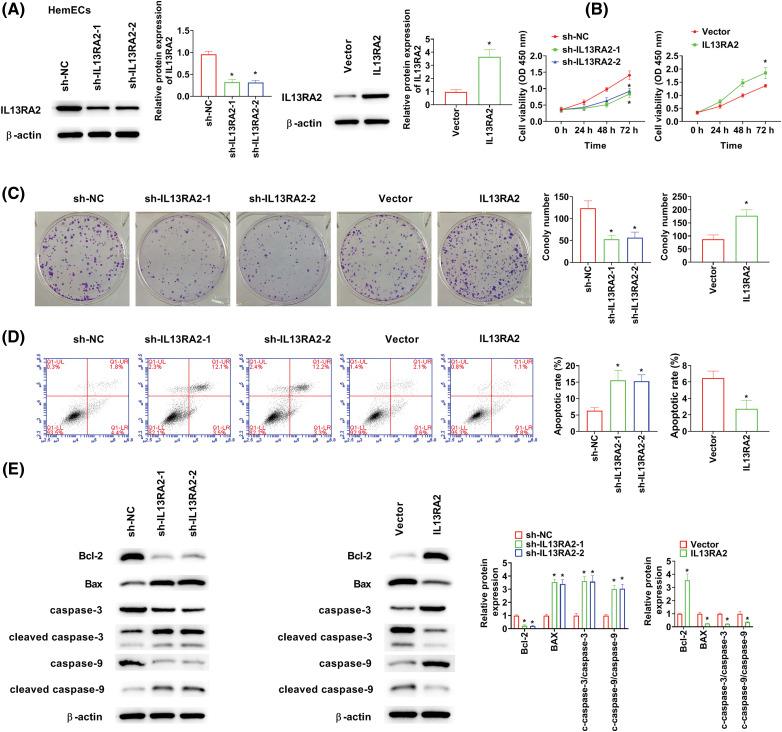
IL13RA2 enhances proliferation of HemECs. (A) Protein expression of IL13RA2 was analyzed by western blot. (B) and (C) Proliferation of HemECs was analyzed by CK-8 and clone formation assays. (D) The results of apoptosis assay. (E) The expression of Bcl-2, Bax, cleaved caspase-3, caspase-3, cleaved caspase-9, and caspase-9 in HemECs was determined by western blot. *p < 0.05.

HemECs were transfected with shIL13RA2-1, shIL13RA2-2, and pcDNA3.1-IL13RA2 vectors. IL13RA2 expression was remarkably knocked down by shIL13RA2-1 and shIL13RA2-2 and over-expressed by pcDNA3.1-IL13RA2 in HemECs (Fig. 2A). Next, we investigated the role of IL13RA2 in HemEC proliferation. CCK-8 assay showed that cell viability was suppressed by IL13RA2 knockdown in HemECs and enhanced by IL13RA2 overexpression in HemECs (Fig. 2B). Colony formation assay revealed similar results (Fig. 2C). Further, IL13RA2 knockdown markedly promoted HemECs apoptosis, whereas IL13RA2 overexpression significantly suppressed HemECs apoptosis (Fig. 2D). Consistently, we also examined the effect of IL13RA2 knockdown or overexpression on apoptosis-related protein levels in HemECs. Bcl-2 level was suppressed by IL13RA2 knockdown and enhanced by IL13RA2 overexpression. In contrast, Bax, cleaved caspase-3/caspase-3, and cleaved caspase-9/caspase-9 levels were enhanced by IL13RA2 knockdown and suppressed by IL13RA2 overexpression (Fig. 2E).

### IL13RA2 promotes migration, invasion, and tubule formation abilities of HemECs

**Figure 3:**
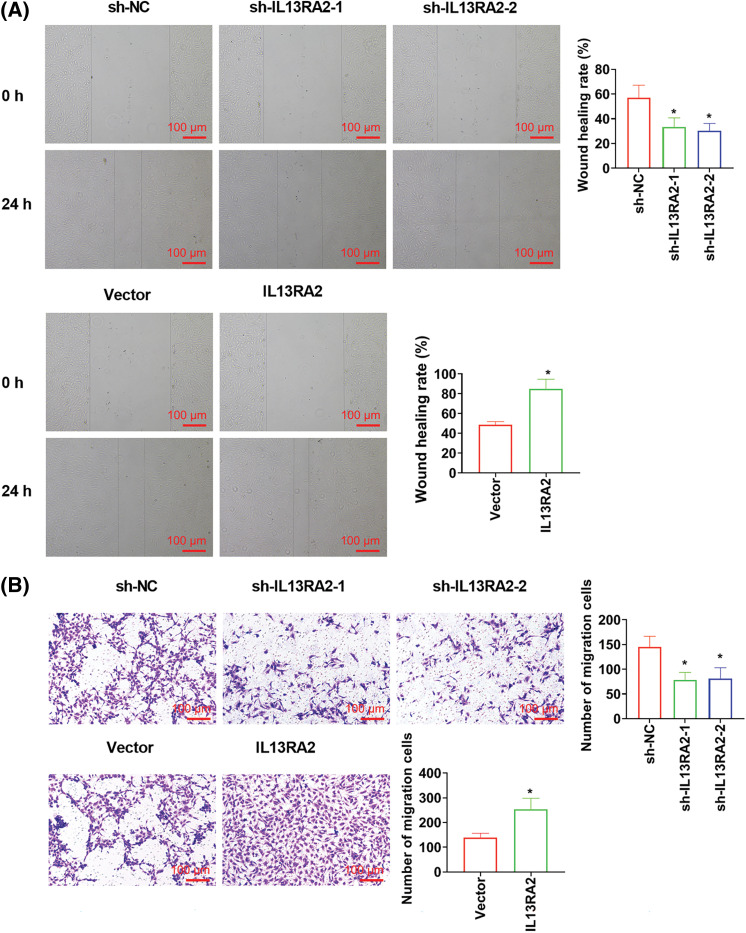

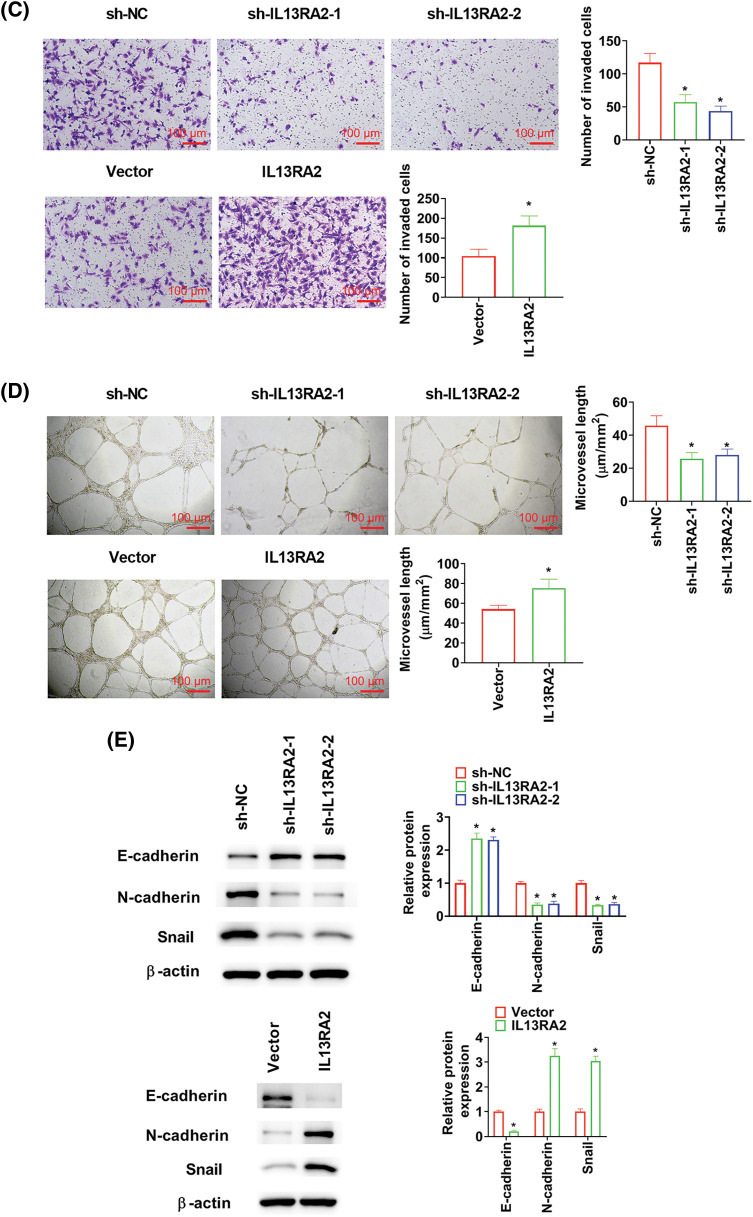
IL13RA2 promotes migration, invasion, and tubules formation abilities of HemECs. (A) Cell migration was detected by wound healing assay. (B) and (C) Cell migration and invasion were measured by Transwell. (D) Tubules formation in HemECs. (E) E-cadherin, Ncadherin, and Snail expression were detected by western blot. *p < 0.05.

IL13RA2 knockdown markedly suppressed cell migration, invasion, and tubules formation abilities of HemECs. While IL13RA2 overexpression promoted cell migration, invasion, and tubules formation abilities of HemECs (Figs. 3A–3D). We also examined EMT-related protein levels after IL13RA2 knockdown or overexpression in HemECs. IL13RA2 knockdown elevated E-cadherin expression, but lightened N-cadherin and Snail expression. IL13RA2 overexpression showed the opposite result (Fig. 3E).

### IL13RA2 activates glycolysis

**Figure 4:**
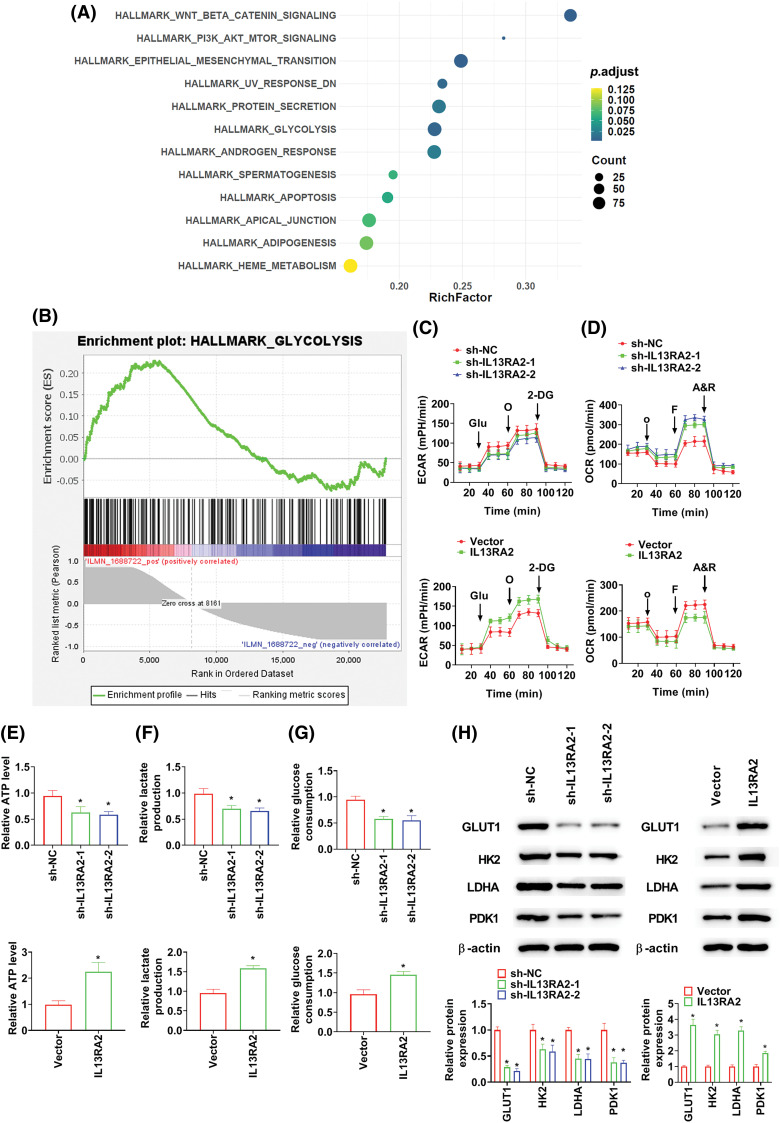

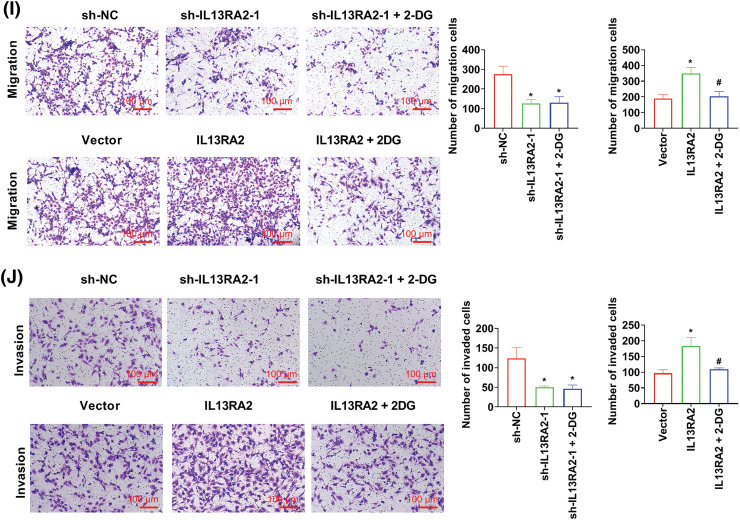
IL13RA2 activates glycolysis. (A) GSEA enrichment analysis. (B) Enrichment plots from GSEA. ECAR (C) and OCR (D) were measured using the Seahorse XF24 analyzer. (E) ATP production in HemECs. (F) Lactate production in HemECs. (G) Glucose consumption in HemECs. (H) The expression of GLUT1, HK2, LDHA, and PDK1 in HemECs was determined by western blot. (I) and (J) Cell migration and invasion were measured by Transwell. *p < 0.05, #*p* < 0.05.

Through GESA, IL13RA2 was found to induce glycolyses in IH (Figs. 4A and 4B). Aggravated glycolysis has been recognised in malignant phenotypes of tumor cells [18]. Therefore, we investigated the impact of IL13RA2 on glycolysis in HemECs. We found that ECAR was declined in sh-IL13RA2-transfected cells and increased in IL13RA2 vector-transfected cells (Fig. 4C). Additionally, The OCR of cells was enhanced by sh-IL13RA2 and decreased by IL13RA2 vector (Fig. 4D). IL13RA2 knockdown decreased ATP, lactate, and glucose consumption levels; whereas IL13RA2 overexpression increased ATP, lactate, and glucose consumption levels (Figs. 4E–4G). To further assess the effects of IL13RA2 on glycolysis, we investigated key glycolytic kinases levels. Protein levels of GLUT1, HK2, LDHA, and PDK1 were inhibited by IL13RA2 knockdown and enhanced by IL13RA2 overexpression (Fig. 4H). Next, we explored whether activation of glycolysis by IL13RA2 facilitates cell migration and invasion. HemECs were treated with 2-DG (a glycolysis inhibitor) at 5 mM for 24 h. In IL13RA2-knockdown cells, 2-DG did not alter cell migration and invasion. IL13RA2 overexpression induced migration and invasion were reversed by 2-DG (Figs. 4I and 4J).

### IL13RA2 activates the Wnt/β-catenin pathway

**Figure 5:**
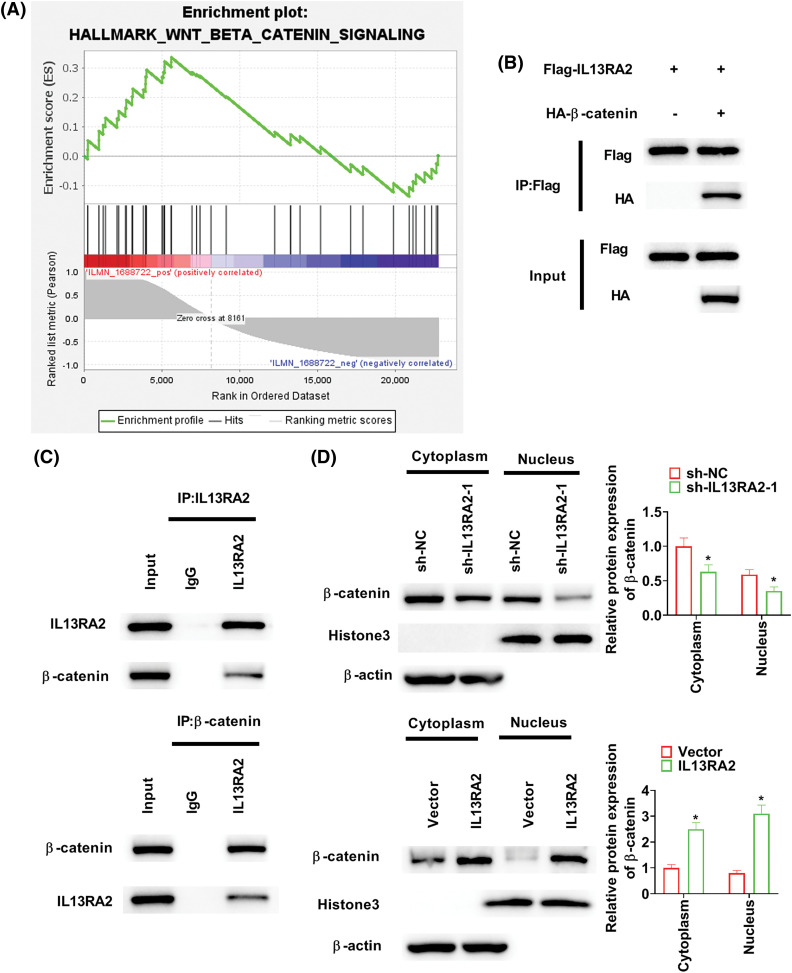

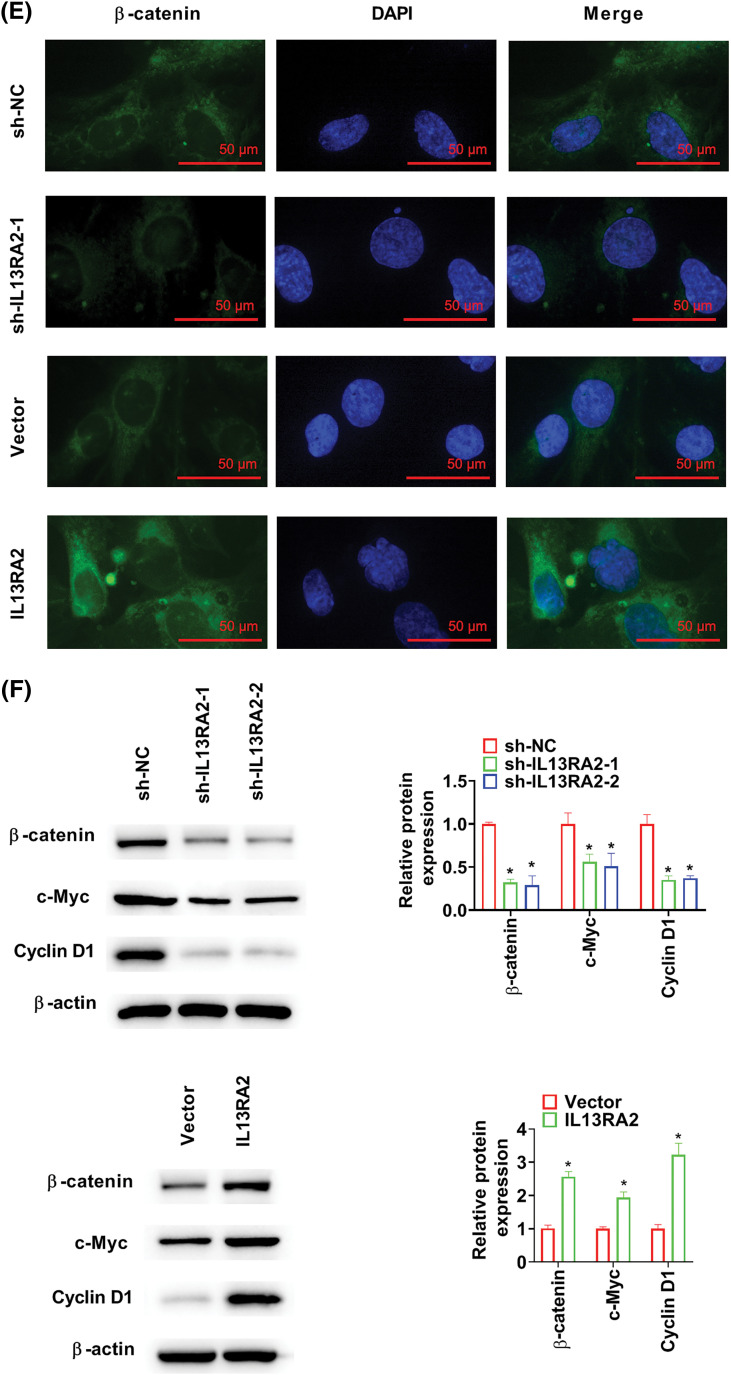
IL13RA2 activates the Wnt/β-catenin pathway. (A) Enrichment plots from GSEA. (B) The interaction between exogenously expressed IL13RA2 and β-catenin in HEK293 cells. (C) Co-IP assays of endogenous IL13RA2 interacted with β-catenin in HemECs. (D) Cytoplasmic and nuclear β-catenin in HemECs. (E) β-catenin expression in HemECs was determined by immunofluorescence. (F) The expression of β-catenin, c-Myc, cyclin D1 in HemECs was detected by western blot. *p < 0.05.

GSEA revealed that the “Wnt/β-catenin signaling” was enriched in HemECs with high IL13RA2 expression (Fig. 5A). To examine how IL13RA2 modulates β-catenin, we performed Co-IP assay. Co-IP analysis in HEK293 and HemECs showed interaction between exogenous and endogenous IL13RA2 and β-catenin (Figs. 5B and 5C). Next, we analysed β-catenin levels in nuclear and cytoplasmic fractions by western blot and immunofluorescence. β-catenin levels in nuclear and cytoplasmic fractions were downregulated by IL13RA2 knockdown and upregulated by IL13RA2 overexpression (Fig. 5D). Additionally, nuclear translocation of β-catenin was reduced in IL13RA2-silenced cells and elevated in IL13RA2-overexpressed cells (Fig. 5E). Next, we detected the levels of downstream genes, c-Myc and cyclinD1. The expression of β-catenin, c-Myc, and cyclin D1 was decreased by IL13RA2 knockdown and increased by IL13RA2 overexpression (Fig. 5F).

### IL13RA2 promotes migration, invasion, and glycolysis through the Wnt/β-catenin pathway

**Figure 6:**
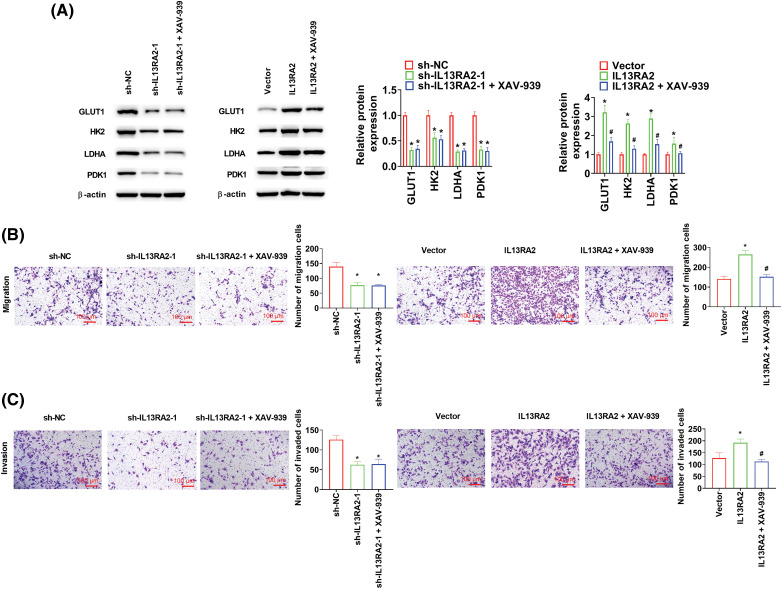
L13RA2 promotes migration, invasion, and glycolysis through Wnt/β-catenin pathway. (A) The expression of GLUT1, HK2, LDHA, and PDK1 in HemECs was detected by western blot. (B) and (C) Cell migration and invasion were measured by Transwell. *p < 0.05, ^#^*p* < 0.05.

HemECs were treated with XAV-939 (a Wnt/β-catenin pathway inhibitor) at 10 μM for 24 h. In IL13RA2 knockdown cells, XAV-939 did not alter the expression of GLUT1, HK2, LDHA, and PDK1, while XAV-939 reversed the increased GLUT1, HK2, LDHA, and PDK1 expression induced by IL13RA2 overexpression (Fig. 6A). Furthermore, XAV-939 did not rescue migration and invasion capacities of IL13RA2-silencing HemECs, but reversed the enhanced migration and invasion capacities of IL13RA2-overexpressing cells (Figs. 6B and 6C).

## Discussion

Although most cases of IH are benign, it can also result in serious complications, such as functional damage and disfigurement [19]. Therefore, the discovery of novel therapeutic targets for IH is necessary. More and more studies have found that IL13RA2 expression is related with tumor progression. IL13RA2 is enhanced in multitudinous patients with glioblastoma [20]. Targeting IL13RA2 is a potential therapeutic approach for glioblastoma [21]. Plasma IL13RA2 levels are linked to overall survival of patients with glioblastoma [22]. In glioblastoma, high IL13RA2 expression predicts worse prognosis [23]. IL13RA2 overexpression was related to poor survival of breast cancer brain metastases [24] and promoted migration of thyroid cancer cells [9]. IL13RA2 knockdown confered invasive and metastatic abilities to hepatocellular carcinoma cells throng activating ERK pathway [25]. In our study, we analysed GSE43742 dataset and found that IL13RA2 expression was remarkably higher in HemECs than that in HDMVECs. Analogously, Mao et al. revealed that CASZ1 expression is enhanced in glioma by analysing GSE22891 and GSE21354 [26]. Small interfering RNA and shRNA have been used widely to silence gene expression in cells [27,28]. In our study, pcDNA3.1-IL13RA2 or shIL13RA2 was transfected to HemECs to overexpress or silence IL13RA2, respectively. IL13RA2 expression was remarkably increased in IH tissues. IL13RA2 overexpression promoted proliferative, migration, invasion, and tubule formation capacities of HemECs. Our results were consistent with a previous study that reported that OIP5-AS1 knockdown halts the proliferative, migration, invasion, and angiogenetic capacities of HemECs [29].

Tumor metabolism primarily relies on aerobic glycolysis [30]. Tumor cells consume energy from aerobic glycolysis to meet their proliferative needs [31]. Through aerobic glycolysis, tumor cells produce ATP and lactate through abundant glucose uptake [32]. Cancer cells membrane has high levels of GLUT1 to excessively uptake more glucose into cells [33]. Downregulation of GLUT1 inhibits glycolysis of tumor cells [34]. HK2, LDHA, and PDK1 are markers of aerobic glycolysis [35]. Chen et al. confirmed that expression of GLUT1, HK2, PFKFB3, PKM2, and LDHA is increased in HemECs [36]. Zheng et al. conformed that PDK1 knockdown observably declines the proliferation and invasion of mouse hemangioendothelioma endothelial (EOMA) cells [37]. Mei et al. indicated that LncRNA-MCM3AP-AS1 promotes aggressiveness of IH by activating glycolysis through modulating miR-138-5p/HIF-1α axis [38]. In our study, IL13RA2 knockdown decreased ATP, lactate, glucose consumption, and GLUT1, HK2, LDHA, and PDK1 expression levels. IL13RA2 overexpression had the opposite effects. A rescue experiment with 2-DG showed that IL13RA2 overexpression induced migration and invasion were reversed with 2-DG treatment. Collectively, these findings suggest that IL13RA2 activates glycolysis in HemECs.

The Wnt/β-catenin pathway is a common oncogenic pathway that is activated in various cancers [14,39]. β-catenin transfer into the nucleus is a key components of the Wnt pathway, leads to the upregulation or downregulation of specific genes [40]. Thomann et al. have reported that low blood vessel density and β-catenin levels in regressed hepatic haemangioma [41]. Ilan et al. demonstrated that nuclear β-catenin is decreased in VEGF-knockdown EOMAcells and increased in VEGF-overexpressed human umbilical vein endothelial cells [42]. Similarly, in our study, β-catenin levels in nuclear and cytoplasmic fractions were downregulated by IL13RA2 knockdown and upregulated by IL13RA2 overexpression. Cyclin D1 and c-Myc are downstream oncogenes of the Wnt/β-catenin pathway [43]. Our data confirmed that β-catenin, c-Myc, and cyclin D1 expression were decreased following IL13RA2 knockdown and increased following IL13RA2 overexpression. A rescue experiment with XAV-939 indicated that the effect of IL13RA2 on IH progression was mediated via the Wnt/β-catenin pathway. Collectively, these findings suggest that IL13RA2 promotes malignant progression of IH by activating the Wnt/β-catenin pathway.

The Wnt/β-catenin pathway is involved in aerobic glycolysis of tumor cells, including nasopharyngeal carcinoma and glioma [44–46]. In colon cancer, activation of the Wnt pathway reportedly increases glycolysis and cell proliferation [47]. PDK1 is pivotal for proliferation of nasopharyngeal cancer cells via the Wnt/β-Catenin pathway [48]. Autophagy-stimulated glycolysis is linked to the Wnt/β-catenin pathway [49]. In hepatocellular carcinoma, PGC1α suppresses aerobic glycolysis via suppressing WNT/β-catenin/PDK1 axis [50]. In lung cancer, DSTYK silencing enhances glycolysis by the Wnt/β-catenin/LDHA axis [51]. In gastric cancer, LiCl, a Wnt/β-catenin pathway activator, reverses the inhibitory effect of butyrate on aerobic glycolysis [52]. In our study, XAV-939 reversed the increase in GLUT1, HK2, LDHA, and PDK1 expression induced by IL13RA2 overexpression, suggesting that IL13RA2 enhances glycolysis of IH by activating the Wnt/β-catenin pathway.

The mechanism of IL13RA2 activation of glycolysis and Wnt/β-catenin pathway affecting HemECs migration and invasion is not clear and in-depth. Further studies will address these limitations.

## Conclusions

IL13RA2 promotes the progression of IH by activating glycolysis and the Wnt/β-catenin pathway. IL13RA2 is a potential therapeutic target for IH.

## Data Availability

The datasets used and analyzed during the current study are available from the corresponding author on reasonable request.

## References

[1] Rodríguez Bandera, A. I., Sebaratnam, D. F., Wargon, O., Wong, L. F. (2021). Infantile hemangioma. Part 1: Epidemiology, pathogenesis, clinical presentation and assessment. Journal of the American Academy of Dermatology*,* 85 *(*6*),* 1379–1392. 10.1016/j.jaad.2021.08.019; 34419524

[2] Hasbani, D. J., Hamie, L. (2022). Infantile hemangiomas. Dermatologic Clinics*,* 40 *(*4*),* 383–392. 10.1016/j.det.2022.06.004; 36243426

[3] Al-Haddad, C., El Salloukh, N. A., El Moussawi, Z. (2019). β-blockers in the treatment of periocular infantile hemangioma. Current Opinion in Ophthalmology*,* 30 *(*5*),* 319–325. 10.1097/ICU.0000000000000591; 31394556

[4] Chinnadurai, S., Sathe, N. A., Surawicz, T. (2016). Laser treatment of infantile hemangioma: A systematic review. Lasers in Surgery and Medicine*,* 48 *(*3*),* 221–233. 10.1002/lsm.v48.3. 26711436

[5] Fei, Q., Lin, Y., Chen, X. (2020). Treatments for infantile hemangioma: A systematic review and network meta-analysis. eClinicalMedicine*,* 26*,* 100506. 10.1016/j.eclinm.2020.100506; 33089121 PMC7565185

[6] Ames, J. A., Sykes, J. M. (2015). Current trends in medical management of infantile hemangioma. Current Opinion in Otolaryngology & Head and Neck Surgery*,* 23 *(*4*),* 286–291. 10.1097/MOO.0000000000000170; 26101875

[7] Ulzii, D., Kido-Nakahara, M., Nakahara, T., Tsuji, G., Furue, K. et al. (2019). Scratching counteracts IL-13 signaling by upregulating the decoy receptor IL-13Rα2 in keratinocytes. International Journal of Molecular Sciences*,* 20 *(*13*),* 3324. 10.3390/ijms20133324; 31284553 PMC6651282

[8] He, B., Liang, J., Qin, Q., Zhang, Y., Shi, S. et al. (2023). IL-13/IL-13RA2 signaling promotes colorectal cancer stem cell tumorigenesis by inducing ubiquitinated degradation of p53. Genes and Diseases*,* 11 *(*1*),* 495–508; 37588218 10.1016/j.gendis.2023.01.027 PMC10425805

[9] Chong, S. T., Tan, K. M., Kok, C. Y. L., Guan, S. P., Lai, S. H. et al. (2019). IL13RA2 is differentially regulated in papillary thyroid carcinoma vs follicular thyroid carcinoma. The Journal of Clinical Endocrinology and Metabolism*,* 104 *(*11*),* 5573–5584. 10.1210/jc.2019-00040; 31290966

[10] Rossmeisl, J. H., Herpai, D., Quigley, M., Cecere, T. E., Robertson, J. L. et al. (2021). Phase I trial of convection-enhanced delivery of IL13RA2 and EPHA2 receptor targeted cytotoxins in dogs with spontaneous intracranial gliomas. Neuro-Oncology*,* 23 *(*3*),* 422–434. 10.1093/neuonc/noaa196; 32812637 PMC7992889

[11] Xie, Y., Wang, M., Xia, M., Guo, Y., Zu, X. et al. (2022). Ubiquitination regulation of aerobic glycolysis in cancer. Life Sciences*,* 292*,* 120322. 10.1016/j.lfs.2022.120322; 35031261

[12] Yang, K., Qiu, T., Zhou, J., Gong, X., Zhang, X. et al. (2023). Blockage of glycolysis by targeting PFKFB3 suppresses the development of infantile hemangioma. Journal of Translational Medicine*,* 21 *(*1*),* 023–03932. 10.1186/s12967-023-03932-y PMC9901151 36740704

[13] Li, M., Wang, X., Yang, E., Li, Y., Geng, Y. et al. (2023). OTUB1 catalytic-independently deubiquitinates TGFBI and mediates the angiogenesis in infantile hemangioma by regulating glycolysis. Arteriosclerosis, Thrombosis, and Vascular Biology*,* 43 *(*5*),* 654–673. 10.1161/ATVBAHA.123.319177; 36994729

[14] Krishnamurthy, N., Kurzrock, R. (2018). Targeting the Wnt/beta-catenin pathway in cancer: Update on effectors and inhibitors. Cancer Treatment Reviews*,* 62*,* 50–60. 10.1016/j.ctrv.2017.11.002; 29169144 PMC5745276

[15] van Schaijik, B., Tan, S. T., Marsh, R. W., Itinteang, T. (2019). Expression of (pro)renin receptor and its effect on endothelial cell proliferation in infantile hemangioma. Pediatric Research*,* 86 *(*2*),* 202–207. 10.1038/s41390-019-0430-8; 31091531

[16] Dai, Y., Zheng, H., Liu, Z., Wang, Y., Hu, W. (2021). The flavonoid luteolin suppresses infantile hemangioma by targeting FZD6 in the Wnt pathway. Investigational New Drugs*,* 39 *(*3*),* 775–784. 10.1007/s10637-020-01052-8; 33411210

[17] Boye, E., Yu, Y., Paranya, G., Mulliken, J. B., Olsen, B. R. et al. (2001). Clonality and altered behavior of endothelial cells from hemangiomas. The Journal of Clinical Investigation*,* 107 *(*6*),* 745–752. 10.1172/JCI11432; 11254674 PMC208946

[18] Chelakkot, C., Chelakkot, V. S., Shin, Y., Song, K. (2023). Modulating glycolysis to improve cancer therapy. International Journal of Molecular Sciences*,* 24 *(*3*),* 2606. 10.3390/ijms24032606; 36768924 PMC9916680

[19] Xu, M. N., Zhang, M., Xu, Y., Wang, M., Yuan, S. M. (2018). Individualized treatment for infantile hemangioma. The Journal of Craniofacial Surgery*,* 29 *(*7*),* 1876–1879. 10.1097/SCS.0000000000004745; 30052610

[20] Debinski, W., Dickinson, P., Rossmeisl, J. H., Robertson, J., Gibo, D. M. (2013). New agents for targeting of IL-13RA2 expressed in primary human and canine brain tumors. PLoS One*,* 8 *(*10*),* e77719. 10.1371/journal.pone.0077719; 24147065 PMC3797726

[21] Sattiraju, A., Solingapuram Sai, K. K., Xuan, A., Pandya, D. N., Almaguel, F. G. et al. (2017). IL13RA2 targeted alpha particle therapy against glioblastomas. Oncotarget*,* 8 *(*26*),* 42997–43007. 10.18632/oncotarget.v8i26. 28562337 PMC5522122

[22] Khristov, V., Nesterova, D., Trifoi, M., Clegg, T., Daya, A. et al. (2022). Plasma IL13Rα2 as a novel liquid biopsy biomarker for glioblastoma. Journal of Neuro-Oncology*,* 160 *(*3*),* 743–752. 10.1007/s11060-022-04196-0; 36436150

[23] Zeng, J., Zhang, J., Yang, Y. Z., Wang, F., Jiang, H. et al. (2020). IL13RA2 is overexpressed in malignant gliomas and related to clinical outcome of patients. American Journal of Translational Research*,* 12 *(*8*),* 4702–4714; 32913543 PMC7476143

[24] Márquez-Ortiz, R. A., Contreras-Zárate, M. J., Tesic, V., Alvarez-Eraso, K. L. F., Kwak, G. et al. (2021). IL13Rα2 promotes proliferation and outgrowth of breast cancer brain metastases. Clinical Cancer Research*,* 27 *(*22*),* 6209–6221. 10.1158/1078-0432.CCR-21-0361; 34544797 PMC8595859

[25] Wang, M., Yao, R., Wang, Y. (2020). Silencing of IL13RA2 promotes partial epithelial-mesenchymal transition in hepatocellular carcinoma via ERK signaling pathway activation. FEBS Open Bio*,* 10 *(*2*),* 229–236. 10.1002/feb4.v10.2. PMC6996351 31823484

[26] Mao, C., Huang, C., Hu, Z., Qu, S. (2020). Transcription factor CASZ1 increases an oncogenic transcriptional process in tumorigenesis and progression of glioma cells. MedComm*,* 3 *(*4*),* e182. 10.1002/mco2.182 PMC9583698 36276925

[27] Wang, H., Zhang, S., Lv, J., Cheng, Y. Y. (2021). Design of polymers for siRNA delivery: Recent progress and challenges. View*,* 2 *(*3*),* 20200026. 10.1002/viw2.v2.3.

[28] Moore, C. B., Guthrie, E. H., Huang, M. T., Taxman, D. J. (2010). Short hairpin RNA (shRNA): Design, delivery, and assessment of gene knockdown. Methods in Molecular Biology*,* 629*,* 141–158; 20387148 10.1007/978-1-60761-657-3_10 PMC3679364

[29] Zhang, J., Zhao, T., Tian, L., Li, Y. (2019). LncRNA OIP5-AS1 promotes the proliferation of hemangioma vascular endothelial cells via regulating miR-195-5p/NOB1 Axis. Frontiers in Pharmacology*,* 10*,* 449. 10.3389/fphar.2019.00449; 31068824 PMC6491816

[30] Vander Heiden, M. G., Cantley, L. C., Thompson, C. B. (2009). Understanding the warburg effect: The metabolic requirements of cell proliferation. Science*,* 324 *(*5930*),* 1029–1033. 10.1126/science.1160809; 19460998 PMC2849637

[31] Zhao, H., Li, Y. (2021). Cancer metabolism and intervention therapy. Molecular Biomedicine*,* 2 *(*1*),* 020–00012. 10.1186/s43556-020-00012-1 PMC8607959 35006438

[32] Schiliro, C., Firestein, B. L. (2021). Mechanisms of metabolic reprogramming in cancer cells supporting enhanced growth and proliferation. Cells*,* 10 *(*5*),* 1056. 10.3390/cells10051056; 33946927 PMC8146072

[33] Kim, I., Kwon, D., Lee, D., Lee, G., Yoon, D. S. (2019). Permselective glucose sensing with GLUT1-rich cancer cell membranes. Biosensors and Bioelectronics*,* 135*,* 82–87. 10.1016/j.bios.2019.04.007; 31004924

[34] Jiang, X., Deng, X., Wang, J., Mo, Y., Shi, L. et al. (2022). BPIFB1 inhibits vasculogenic mimicry via downregulation of GLUT1-mediated H3K27 acetylation in nasopharyngeal carcinoma. Oncogene*,* 41 *(*2*),* 233–245. 10.1038/s41388-021-02079-8; 34725462

[35] Zhang, Z., Zheng, Y., Chen, Y., Yin, Y., Chen, Q. et al. (2022). Gut fungi enhances immunosuppressive function of myeloid-derived suppressor cells by activating PKM2-dependent glycolysis to promote colorectal tumorigenesis. Experimental Hematology & Oncology*,* 11 *(*1*),* 022–00334. 10.1186/s40164-022-00334-6 PMC9644472 36348389

[36] Chen, J., Wu, D., Dong, Z., Chen, A., Liu, S. (2020). The expression and role of glycolysis-associated molecules in infantile hemangioma. Life Sciences*,* 259*,* 118215. 10.1016/j.lfs.2020.118215; 32768579

[37] Zheng, N., Ding, X., Sun, A., Jahan, R. (2015). PDK1 activity regulates proliferation, invasion and growth of hemangiomas. Cellular Physiology and Biochemistry*,* 36 *(*5*),* 1903–1910. 10.1159/000430159; 26202351

[38] Mei, H., Xian, H., Ke, J. (2021). LncRNA-MCM3AP-AS1 promotes the progression of infantile hemangiomas by increasing miR-138-5p/HIF-1α axis-regulated glycolysis. Frontiers in Molecular Biosciences*,* 8*,* 753218. 10.3389/fmolb.2021.753218; 34660700 PMC8511435

[39] Manni, W., Min, W. (2020). Signaling pathways in the regulation of cancer stem cells and associated targeted therapy. MedComm*,* 3 *(*4*),* e176. 10.1002/mco2.176 PMC9534377 36226253

[40] Anthony, C. C., Robbins, D. J., Ahmed, Y., Lee, E. (2020). Nuclear regulation of Wnt/β-catenin signaling: It’s a complex situation. Genes*,* 11 *(*8*),* 886. 10.3390/genes11080886; 32759724 PMC7465203

[41] Thomann, S., Tóth, M., Sprengel, S. D., Liermann, J., Schirmacher, P. (2022). Digital staging of hepatic hemangiomas reveals spatial heterogeneity in endothelial cell composition and vascular senescence. The Journal of Histochemistry and Cytochemistry*,* 70 *(*7*),* 531–541. 10.1369/00221554221112701; 35815421 PMC9284234

[42] Ilan, N., Tucker, A., Madri, J. A. (2003). Vascular endothelial growth factor expression, beta-catenin tyrosine phosphorylation, and endothelial proliferative behavior: A pathway for transformation? Laboratory Investigation*,* 83 *(*8*),* 1105–1115. 10.1097/01.LAB.0000083531.84403.8B; 12920240

[43] Zhong, M., Zhou, L., Fang, Z., Yao, Y. Y., Zou, J. P. et al. (2021). Ubiquitin-specific protease 15 contributes to gastric cancer progression by regulating the Wnt/β-catenin signaling pathway. World Journal of Gastroenterology*,* 27 *(*26*),* 4221–4235. 10.3748/wjg.v27.i26.4221; 34326621 PMC8311539

[44] Cai, C. F., Ye, G. D., Shen, D. Y., Zhang, W., Chen, M. L. et al. (2018). Chibby suppresses aerobic glycolysis and proliferation of nasopharyngeal carcinoma via the Wnt/β-catenin-Lin28/let7-PDK1 cascade. Journal of Experimental & Clinical Cancer Research*,* 37 *(*1*),* 104. 10.1186/s13046-018-0769-4; 29764469 PMC5952826

[45] Vallée, A., Guillevin, R., Vallée, J. N. (2018). Vasculogenesis and angiogenesis initiation under normoxic conditions through Wnt/β-catenin pathway in gliomas. Reviews in the Neurosciences*,* 29 *(*1*),* 71–91. 28822229 10.1515/revneuro-2017-0032

[46] Vallée, A., Lecarpentier, Y., Vallée, J. N. (2021). The key role of the WNT/β-catenin pathway in metabolic reprogramming in cancers under normoxic conditions. Cancers*,* 13 *(*21*),* 5557. 10.3390/cancers13215557; 34771718 PMC8582658

[47] Pate, K. T., Stringari, C., Sprowl-Tanio, S., Wang, K., TeSlaa, T. et al. (2014). Wnt signaling directs a metabolic program of glycolysis and angiogenesis in colon cancer. EMBO Journal*,* 33 *(*13*),* 1454–1473. 10.15252/embj.201488598; 24825347 PMC4194089

[48] Cai, C. F., Ye, G. D., Shen, D. Y., Zhang, W., Chen, M. L. et al. (2018). Chibby suppresses aerobic glycolysis and proliferation of nasopharyngeal carcinoma via the Wnt/β-catenin-Lin28/let7-PDK1 cascade. Journal of Experimental & Clinical Cancer Research*,* 37 *(*1*),* 018–0769. 10.1186/s13046-018-0769-4 PMC5952826 29764469

[49] Fan, Q., Yang, L., Zhang, X., Ma, Y., Li, Y. et al. (2018). Autophagy promotes metastasis and glycolysis by upregulating MCT1 expression and Wnt/β-catenin signaling pathway activation in hepatocellular carcinoma cells. Journal of Experimental & Clinical Cancer Research*,* 37 *(*1*),* 018–0673. 10.1186/s13046-018-0673-y PMC5775607 29351758

[50] Zuo, Q., He, J., Zhang, S., Wang, H., Jin, G. et al. (2021). PPARγ coactivator-1α Suppresses metastasis of hepatocellular carcinoma by inhibiting warburg effect by PPARγ-dependent WNT/β-catenin/pyruvate dehydrogenase kinase isozyme 1 axis. Hepatology*,* 73 *(*2*),* 644–660. 10.1002/hep.31280; 32298475

[51] Zhong, C., Chen, M., Chen, Y., Yao, F., Fang, W. (2021). Loss of DSTYK activates Wnt/β-catenin signaling and glycolysis in lung adenocarcinoma. Cell Death & Disease*,* 12 *(*12*),* 1122. 10.1038/s41419-021-04385-1; 34853310 PMC8636471

[52] Liang, Y., Rao, Z., Du, D., Wang, Y., Fang, T. (2023). Butyrate prevents the migration and invasion, and aerobic glycolysis in gastric cancer via inhibiting Wnt/β-catenin/c-Myc signaling. Drug Development Research*,* 84 *(*3*),* 532–541; 36782390 10.1002/ddr.22043

